# The Discriminatory Attitudes of Health Workers against People Living with HIV

**DOI:** 10.1371/journal.pmed.0020261

**Published:** 2005-07-19

**Authors:** Gobopamang Letamo

## Abstract

Letamo discusses the implications of a new study suggesting that some health professionals discriminate against people with HIV.

Anecdotal evidence suggests that health-care professionals in Nigeria may discriminate against and stigmatise people living with HIV/AIDS(PLWA). In a study in the August issue of *PLoS Medicine*, Reis and colleagues set out to characterise the nature and extent of discriminatory practices and attitudes in the health sector, and indicate possible contributing factors and intervention strategies [1]. The study was specifically designed to answer three research questions: (1) are there discriminatory practices in the health sector that affect the health and well-being of PLWA in Nigeria, (2) how receptive are health workers and institutions to treating PLWA, and (3) what underlying factors may contribute to any discriminatory practices?

In order to answer these questions, a representative sample of 1,103 health-care professionals (doctors, nurses, and midwives) working directly with patients with HIV/AIDS were selected from four states in Nigeria and asked to participate in a study. The response rate was 93% (i.e., 1,021 surveyed professionals participated). A survey questionnaire was administered to respondents to collect information about their knowledge, attitudes, and behaviour. The study was reviewed and approved by an independent ethics review board of individuals with expertise in clinical medicine, public health, bioethics, and international HIV/AIDS and human rights research.

## Main Findings of the Study

The results suggest that some health-care professionals discriminate against and stigmatise PLWA. For instance, 9% of professionals reported refusing to care for a patient with HIV/AIDS, and 9% reported that they refused a patient with HIV/AIDS admission to hospital. Two-thirds reported observing other health professionals refusing to care for a patient with HIV/AIDS, and 43% observed others refusing a patient with HIV/AIDS admission to hospital.

The study suggests that a significant number of health-care professionals engage in discriminatory and unethical behaviour. Some professionals reported giving confidential information to other people (family members and unrelated individuals) without the patient's consent. Despite these discriminatory attitudes, an optimistic finding is that most health-care professionals expressed an interest in additional information and suggested education and counselling as a way to address discriminatory behaviours by their colleagues.

Myths and misconceptions about HIV transmission play a role in promoting discrimination.

The study concludes that all clinical staff should be educated about HIV/AIDS, modes of transmission of the virus, universal precautions, and the rights of PLWA. Such education is likely to reduce discriminatory practices towards PLWA and may improve these patients' care and access to health services. The study also asserts that a lack of protective materials and other materials needed to treat and prevent the spread of HIV, documented in several health facilities and reported by professionals themselves, contributes to discriminatory behaviour among health professionals.

## Implications of the Study

The study raises the possibility (although does not prove) that patients with HIV/AIDS may not fully utilise health-care services because they are denied access by health-care providers who discriminate against them. The fact that some health workers have discriminated against PLWA in the past suggests that health-care professionals serving PLWA should urgently be educated about HIV/AIDS so that they fully understand how HIV can and cannot be transmitted.

It is clear from Reis and colleagues' study and others, including my own research in Botswana [2–4], that myths and misconceptions about how HIV can and cannot be transmitted play an important role in promoting discrimination. The implication of these findings is clear—there is a dire need to strengthen the information, education, and communication component of HIV/AIDS prevention efforts in order to dispel misconceptions that people tend to hold.

In my own research in Botswana, I found that although the prevalence of discriminatory attitudes towards PLWA may be high, respondents tend to be less discriminating when the HIV-positive person happens to be a family member [2]. People are reluctant to make the serostatus of their relatives public when their relatives are HIV positive, for fear of discrimination.

In order to further our understanding of the root causes of discrimination and stigmatisation of PLWA, qualitative research is needed to understand cognitive processes that lead one to discriminate. It would also be interesting to investigate how PLWA feel that they are perceived by people around them. In order to adequately address issues of discrimination, we must involve PLWA and find out what they feel needs to be done to address stigma and discrimination. We also need to investigate the extent to which researchers are able to measure what they purport to measure with the current indicators of discrimination and stigmatisation. It is possible that the prevalence of stigma and discrimination against PLWA is not being adequately measured with the research instruments currently in use.

## Abbreviation

PLWA: people living with HIV/AIDS
